# Digoxin-induced retinal degeneration depends on rhodopsin

**DOI:** 10.1038/cddis.2017.94

**Published:** 2017-03-16

**Authors:** Britta Landfried, Marijana Samardzija, Maya Barben, Christian Schori, Katrin Klee, Federica Storti, Christian Grimm

**Affiliations:** 1 Lab for Retinal Cell Biology, Department of Ophthalmology, University of Zürich, Zürich, Switzerland; 2 Neuroscience Center Zürich (ZNZ), University of Zürich, Zürich, Switzerland; 3 Center for Integrative Human Physiology (ZIHP), University of Zürich, Zürich, Switzerland

## Abstract

Na,K-ATPases are energy consuming ion pumps that are required for maintaining ion homeostasis in most cells. In the retina, Na,K-ATPases are especially important to sustain the dark current in photoreceptor cells needed for rapid hyperpolarization of rods and cones in light. Cardiac glycosides like digoxin inhibit the activity of Na,K-ATPases by targeting their catalytic alpha subunits. This leads to a disturbed ion balance, which can affect cellular function and survival. Here we show that the treatment of wild-type mice with digoxin leads to severe retinal degeneration and loss of vision. Digoxin induced cell death specifically in photoreceptor cells with no or only minor effects in other retinal cell types. Photoreceptor-specific cytotoxicity depended on the presence of bleachable rhodopsin. Photoreceptors of *Rpe65* knockouts, which have no measurable rhodopsin and photoreceptors of *Rpe65*^*R91W*^ mice that have <10% of the rhodopsin found in retinas of wild-type mice were not sensitive to digoxin treatment. Similarly, cones in the all-cone retina of *Nrl* knockout mice were also not affected. Digoxin induced expression of several genes involved in stress signaling and inflammation. It also activated proteins such as ERK1/2, AKT, STAT1, STAT3 and CASP1 during a period of up to 10 days after treatment. Activation of signaling genes and proteins, as well as the dependency on bleachable rhodopsin resembles mechanisms of light-induced photoreceptor degeneration. Digoxin-mediated photoreceptor cell death may thus be used as an inducible model system to study molecular mechanisms of retinal degeneration.

Retinal degenerative diseases like retinitis pigmentosa or age-related macular degeneration (AMD) lead to strong visual impairment or blindness and have a high socio-economic impact. Although they affect a large number of patients and the incidence is expected to increase during the next decades especially for AMD,^1^ an efficient treatment option is still an unmet medical need. A main reason for the lack of therapies is the insufficient understanding of the pathophysiological mechanisms responsible for disease initiation and progression even though many animal models have been developed and studied.^2^ These models are either based on genetic manipulations or on the application of toxic stimuli such as light^3^ or N-methyl-N-nitrosourea (MNU)^4^ and may give insights into specific aspects of disease pathology. Nevertheless, additional models based on different molecules or pathways are desirable to further broaden approaches and experimental possibilities for investigations.

Cardiac glycosides like digoxin or ouabain inhibit Na,K-ATPases and are traditionally used to treat atrial fibrillation and atrial flutter.^5^ Owing to their cytotoxic properties, they are also discussed as possible anticancer agents.^6^ Although different cardiac glycosides have slightly different specificities for different isoforms of the Na,K-ATPases,^7^ their inhibitory action on the ion pump causes increased intracellular Na^+^ and Ca^2+^ and decreased K^+^ concentrations.^8^ This disturbance of the ion homeostasis may affect cellular function and viability. As cardiac glycosides inhibit Na,K-ATPases in all tissues including the retina, it is not surprising that several reports suggest an effect of digoxin on vision, with strongest disturbances of the cone pathway.^9, 10^

Na,K-ATPases are heterodimers, composed of an alpha- and a beta-subunit.^11^ In addition, they can be regulated by an auxiliary FXYD protein.^12, 13^ Cardiac glycosides target primarily the catalytic alpha subunit of the Na,K-ATPase.^14, 15^ Four alpha subunits have been identified that are encoded by different genes (*Atp1a1*–*Atp1a4*) and show tissue-specific expression. Whereas *α*1 is quite ubiquitously expressed in many tissues including the retina, *α*2 and *α*3 show a more specific pattern and have also been found in brain and retina.^16^ Whereas *α*2 may be specific for Müller glia cells, *α*3 has been identified on all retinal neurons with particularly strong expression in photoreceptors, but not on Müller cells or the retinal pigment epithelium (RPE).^17^ In contrast, *α*4 may be restricted to testis where it is important for sperm maturation.^18^

Since digoxin shows some preference for *α*2 and *α*3 isoforms^7^ it may affect activity of Na,K-ATPases in the retina. Reports of patients describing visual disturbances, blurred vision, central scotomas or seeing yellowish^19^ after digoxin treatment support this conclusion. Similarly, studies in monkeys demonstrated that digoxin can affect function especially of the cone system,^10^ and a recent report showed that treating mice with high doses of digoxin causes retinal degeneration.^20^ Thus, we investigated the effect of digoxin on the mouse retina and focused on its potential cytotoxicity for photoreceptor cells in different wild type and transgenic mouse lines. We established a treatment protocol that induces severe loss of photoreceptors and propose that digoxin-mediated photoreceptor cell death may be used as a novel model to study mechanisms of retinal degeneration.

## Results

### Digoxin-induced retinal degeneration

**Figure 1:**
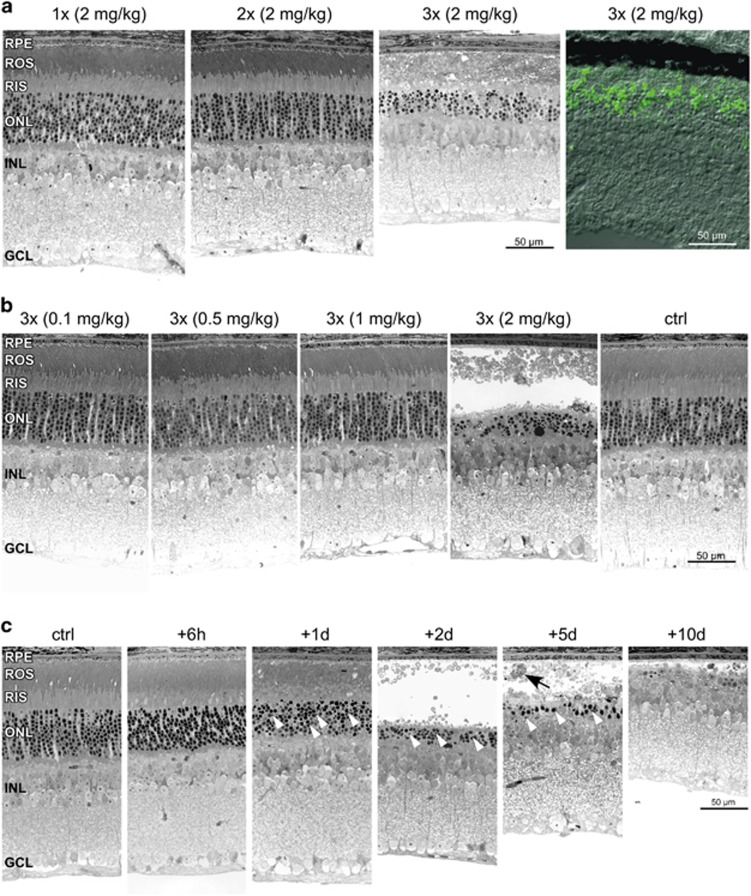
Digoxin-induced retinal degeneration. (**a**) Retinal morphology of mice at 2 days after the last of 1, 2 or 3 injections of 2 mg/kg digoxin as indicated. TUNEL staining (right panel) at 1 day after the last of 3 injections of 2 mg/kg digoxin. (**b**) Retinal morphology of mice at 2 days after the last of 3 injections of 0.1, 0.5, 1 or 2 mg/kg digoxin as indicated. (**c**) Retinal morphology of mice at 6 h, 1 d, 2 d, 5 d and 10 d after the last of 3 injections of 2 mg/kg digoxin as indicated. *N*=3. Black arrow, macrophage; Ctrl, PBS-injected control mice; GCL, ganglion cell layer; INL, inner nuclear layer; ONL, outer nuclear layer; RPE, retinal pigment epithelium; ROS, rod outer segments; RIS, rod inner segments; Scale bars, 50 *μ*m; white arrowheads: pyknotic nuclei

**Figure 2:**
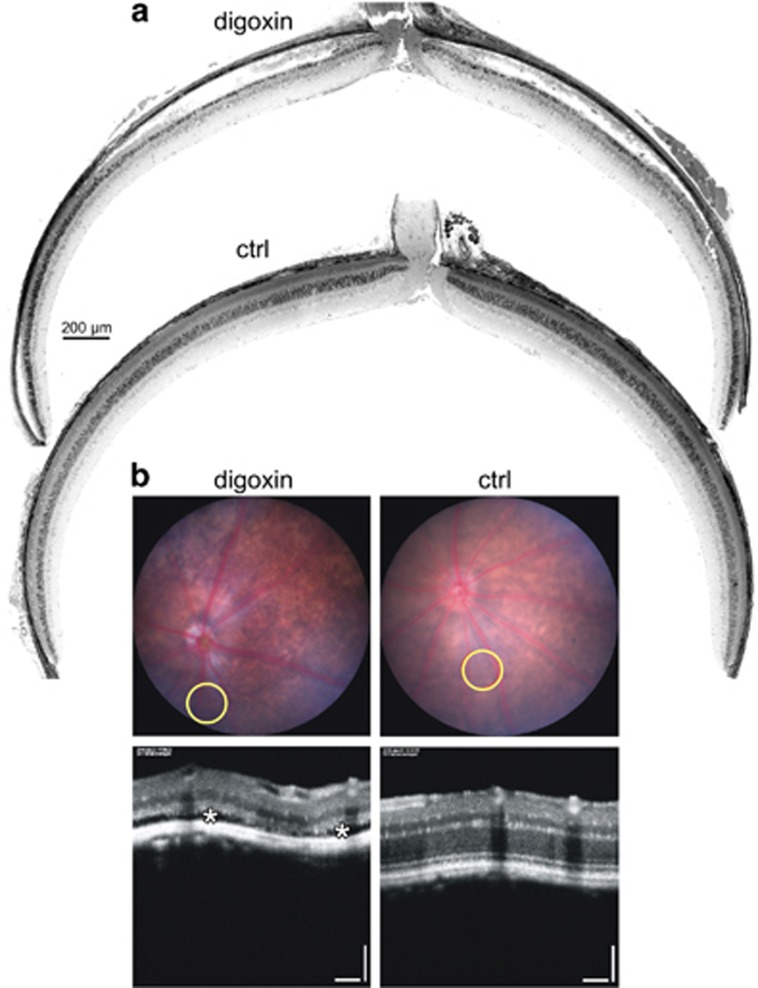
Geographic extent and *in vivo* imaging of digoxin-induced retinal degeneration. (**a**) Dorsoventral retinal panoramas of digoxin- (digoxin, top) or PBS-treated (ctrl, bottom) mice at 2 days after the last of 3 injections of 2 mg/kg digoxin. Scale bar, 200 *μ*m. *N*=3. (**b**) Fundus (top) and OCT (bottom) images of digoxin (digoxin, left) and untreated (ctrl, right) mice at 9 days after the last of 3 injections of 2 mg/kg digoxin. Yellow circles indicate the position of the corresponding OCT images. White asterisks mark subretinal fluid accumulation. Scale bar, 100 *μ*m. *N*=2

To characterize the morphological, functional and molecular response to digoxin treatment, we titrated the amount of digoxin and the number of intraperitoneal (ip) injections needed for the induction of photoreceptor degeneration in C57BL/6 mice. In a first set of experiments, we used a dose of 2 mg/kg that was reported by Hinshaw *et al.*^20^ to cause photoreceptor degeneration after 5 (least number of injections reported) or more intraperitoneal injections. This dose induced severe retinal degeneration already after three daily ip injections whereas one and two injections did not noticeably alter retinal morphology (Figure 1a). Although the extent of degeneration was slightly variable, cell death was largely restricted to the outer nuclear layer (ONL) as almost no TUNEL-positive cells were detected in other retinal layers (Figure 1a). Three injections of a lower dose (up to 1 mg/kg) did not cause degeneration (Figure 1b). Consequently, a treatment protocol of one digoxin injection on each of three consecutive days at a dose of 2 mg/kg was established and used for most of the following experiments. Although strong morphological damage was observed at 2 days, we detected pyknotic nuclei in the ONL as early as 1 day after the last injection (Figure 1c, white arrowheads). This was followed by a complete disruption of the outer and inner segments and loss of photoreceptors in the central retina between 2 and 10 days, the last time point analyzed (Figure 1c). Degeneration was accompanied by macrophage infiltration to the subretinal space (Figure 1c, black arrow). Loss of photoreceptor cells was most significant in the central retina whereas the retinal periphery was largely spared (Figures 2a and 6f). Consequences of digoxin treatment were also followed *in vivo* by fundus photography and optical coherence tomography (OCT) at 9 days after treatment (Figure 2b). Injections of digoxin caused the fundus to appear more intensely pigmented, probably due to the thinned retinal tissue. OCT images confirmed severe thinning of the ONL in digoxin-treated but not in control mice. Some regions of the retina in digoxin-treated mice were detached from the RPE suggesting potential subretinal fluid accumulation (Figure 2b).

**Figure 3:**
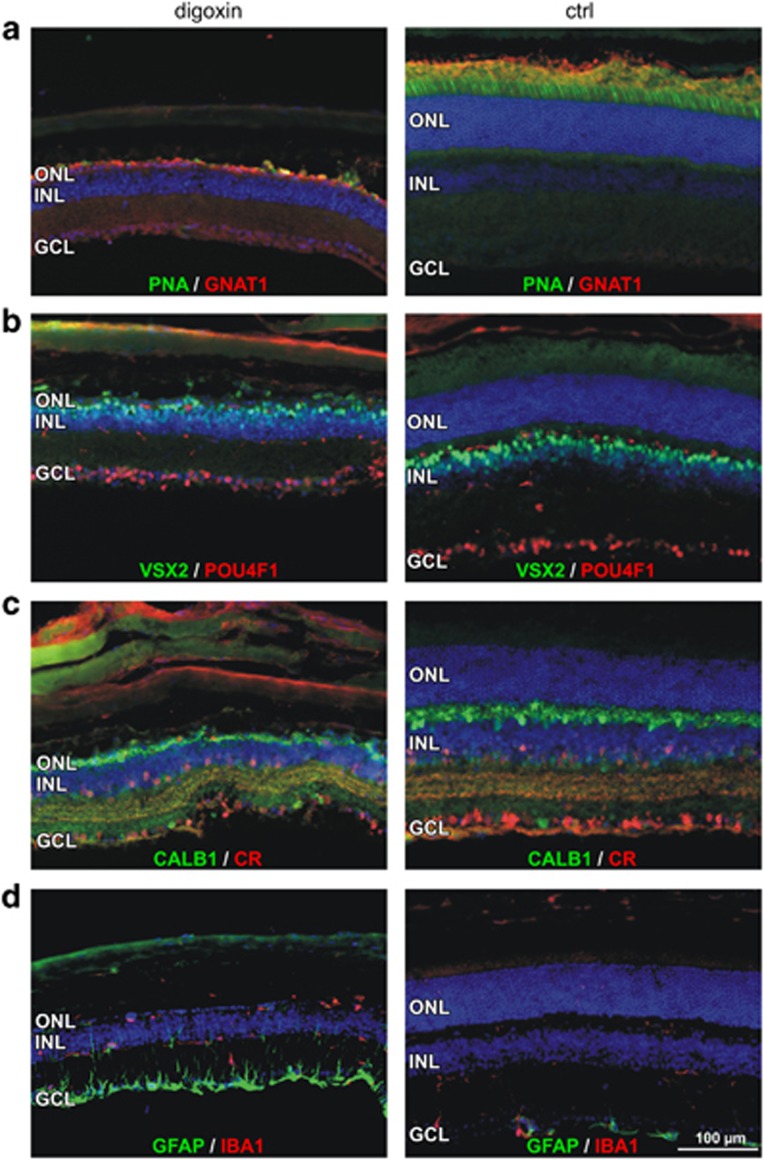
Localization of retinal cell markers after digoxin treatment. Retinal sections of mice at 10 days after the last of 3 injections of 2 mg/kg digoxin (left) or PBS (ctrl, right) were stained with PNA (green) and antibodies against GNAT1 (red) (**a**), VSX2 (green) and POU4F1 (red) (**b**), CALB1 (green) and CR (red) (**c**) and GFAP (green) and IBA1 (red) (**d**). Ctrl, PBS-injected control mice; GCL, ganglion cell layer; INL, inner nuclear layer; ONL, outer nuclear layer; RPE, retinal pigment epithelium; ROS, rod outer segments; RIS, rod inner segments; Scale bars, 100 *μ*m. *N*=3

TUNEL staining (Figure 1a) suggested that the toxic effect of digoxin was largely restricted to photoreceptor cells. This conclusion was further supported by immunofluorescence staining of retinal cell markers in retinas at 10 days after digoxin or PBS treatment. Distribution of peanut agglutinin (PNA) and G-protein subunit alpha transducin 1 (GNAT1) showed that both cones and rods were directly or indirectly affected by digoxin (Figure 3a). In contrast, normal distribution of visual system homeobox 2 (VSX2), pou domain, class4, transcription factor 1 (POU4F1), calbindin 1 (CALB1) and calretinin (CR) suggested that bipolar cells, ganglion cells, horizontal cells and amacrine cells, respectively, were largely unaffected (Figures 3b and c). Nevertheless, digoxin induced degeneration of photoreceptors activated Müller glia cells as indicated by increased GFAP staining and stimulated resident IBA1-positive microglia and/or macrophages to migrate to the subretinal space (Figure 3d).

**Figure 4:**
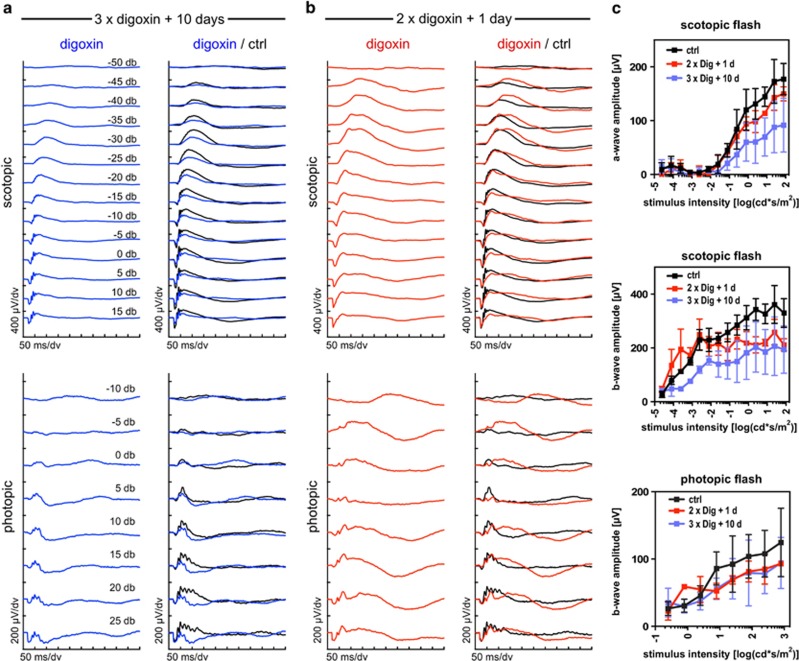
Retinal function after digoxin treatment. Mice received a toxic dose (three injections, 2 mg/kg each) or a subtoxic dose (two injections, 2 mg/kg each) of digoxin and were analyzed at 10 or 1 day after the last injection, as indicated. (**a**) Scotopic (top) and photopic (bottom) ERG traces were recorded from mice treated with the toxic digoxin dose (3 × digoxin, blue lines). (**b**) Scotopic (top) and photopic (bottom) ERG traces were recorded from mice treated with the subtoxic digoxin dose (2 × digoxin, red lines). ERGs were compared to mice injected twice with PBS (ctrl) and analyzed 1 day after the second injection (black lines in **a** and **b**). Shown are traces averaged from *N*=3 mice (6 eyes). (**c**) Scotopic a- and b-wave and photopic b-wave amplitudes plotted as a function of stimulus intensity. Shown are averages±S.D. *N*=3 mice (6 eyes). Color scheme as in (**a** and **b**)

As expected, digoxin-induced loss of photoreceptors affected scotopic and photopic retinal function at 10 days after treatment. Scotopic and photopic b-wave, as well as scotopic a-wave amplitudes were reduced indicating loss of function (Figures 4a and c). Even though morphological and immunofluorescence analyses suggested that digoxin toxicity was confined to photoreceptors in the outer retina, cells of the inner retina may have been affected functionally. ERG recordings one day after two digoxin injections (2 mg/kg), a treatment that did not induce photoreceptor degeneration (Figure 1a), showed prolonged responses at low light intensities, a reduction of the maximal b-wave amplitudes and strongly reduced oscillatory potentials for both the scotopic and the photopic ERG (Figures 4b and c). These observations resemble functional consequences reported for ocular ischemia^21^ that is characterized by a reduced supply of oxygen to the inner retina.^22^ Interestingly, digoxin can reduce heart rate^23^ and cause vasoconstriction of peripheral arteries,^24^ and may thus potentially lead to a reduced blood flow in peripheral organs including the retina. Clearly, further experimentation is required to evaluate the influence of digoxin on retinal function.

### Molecular signaling

**Figure 5:**
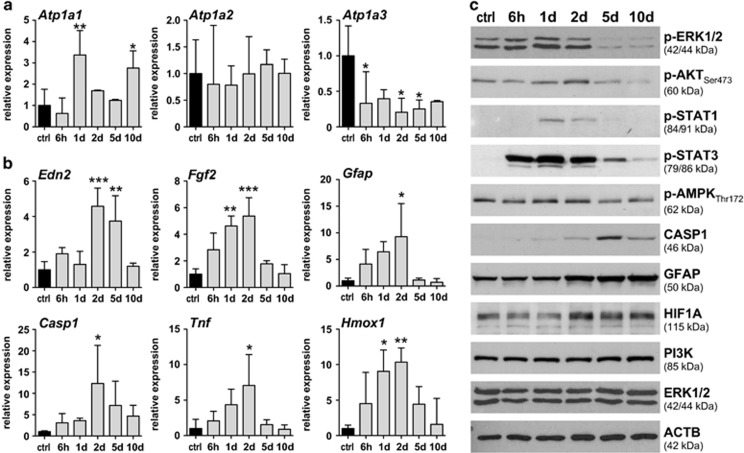
Retinal gene and protein expression after digoxin treatment. (**a**) Relative expression of genes encoding the alpha subunits of Na,K-ATPases. (**b**) Relative expression of genes involved in stress signaling and inflammation, as indicated. Retinal expression levels of digoxin-treated mice (gray bars) were normalized to *Actb* and expressed as fold change relatively to PBS-injected controls (ctrl, black bars, set to 1). Shown are means±S.D. of *N*=3. *: adjusted *P*-value<0.05. **: adjusted *P*-value<0.01. ***: adjusted *P*-value<0.001. One-way ANOVA with Dunnett's multiple comparison test (each column compared to control). (**c**) Retinal levels of selected proteins and phospho-proteins were tested by Western blotting. All analyses were done at five time points (as indicated) after the last of 3 injections of 2 mg/kg digoxin and compared to PBS-injected controls (ctrl). *N*=3

Digoxin inhibits Na,K-ATPases^25^ through interaction with the regulatory alpha subunits, with a preference for *α*2 and *α*3 (ref. 26) (see ‘Introduction' section). As *α*4 was reported to be specifically expressed in testis,^18^ we focused on *α*1, *α*2 and *α*3 for our analysis. As reported before^17^ all three alpha subunits were expressed in the normal mouse retina (Figure 5a). Treatment with digoxin increased expression of *Atp1a1* at 1 d and 10 d, did not affect *Atp1a2* and reduced the expression of *Atp1a3* (Figure 5a). As *α*3 shows a strong expression in photoreceptors whereas *α*1 and *α*2 may be expressed preferentially by other retinal cells,^17^ downregulation of *Atp1a3* expression may reflect the degenerative process with photoreceptor injury and death. In addition, digoxin treatment induced retinal expression of several genes involved in stress signaling such as endothelin-2 (*Edn2*), fibroblast growth factor 2 (*Fgf2*) and glial fibrillary acidic protein (*Gfap*), in degeneration and inflammation including caspase 1 (*Casp1*) and tumor necrosis factor alpha (*Tnf*), and in oxidative stress like heme oxygenase 1 (*Hmox1*; Figure 5b). Peak of activation was at 2 days after the last digoxin injection. Expression levels returned to basal levels at 10 days after injections suggesting a general recovery from treatment. This interpretation was supported by the pattern of protein activation (Figure 5c). Phosphorylation of the signaling proteins extracellular signal regulated kinase (ERK), protein kinase B (alias AKT), signal transducer and activator of transcription 1 (STAT1) and of STAT3 peaked at 1 and 2 days after treatment, reaching basal levels thereafter. Interestingly, we consistently observed that levels of pERK1/2 were strongly reduced at 5 and 10 days post treatment. The reason and the functional consequences for such a reduced pERK signaling are unknown and remain to be evaluated. It has been reported that digoxin increases phosphorylation of protein kinase AMP-activated catalytic subunit alpha 1 (AMPK) and may thus affect energy levels in cells.^27^ In retinas of digoxin-treated mice, however, pAMPK levels remained constant with a slight decrease by 5 and 10 days after the last injection. In contrast, proteins indicating reactive gliosis and degeneration (GFAP and CASP1) were induced primarily between 2 and 10 days, and thus after the initial signaling events. A late response of CASP1 was also observed in other models of retinal degeneration.^28^ As in the light damage model and during degeneration in the VPP mouse model for inherited degeneration,^28^ we did not detect P10 or P20 cleaved forms of CASP1 (not shown). Levels of the p85 regulatory subunit of PI3K remained at constant levels, even though this protein is known to be involved in Na,K-ATPase signaling.^29, 30^ Levels of hypoxia-inducible factor 1A (HIF1A) were bi-phasic with an initial decline at 1 d, followed by an increase until 10 d post treatment. Although ERG data (Figure 4) may indicate that digoxin application resulted in a condition resembling retinal ischemia, we did not detect increased HIF1A levels shortly after treatment presumably because digoxin can inhibit HIF1A, as reported by others.^31, 32^ Only after clearance of digoxin, HIF1A levels may increase and reach the levels detected between 2 and 10 days. However, this bi-phasic appearance of HIF1A was not reflected by corresponding mRNA levels of HIF1 target genes suggesting that HIF1 activity was not strongly affected by digoxin (data not shown).

### Dependency on rhodopsin

**Figure 6:**
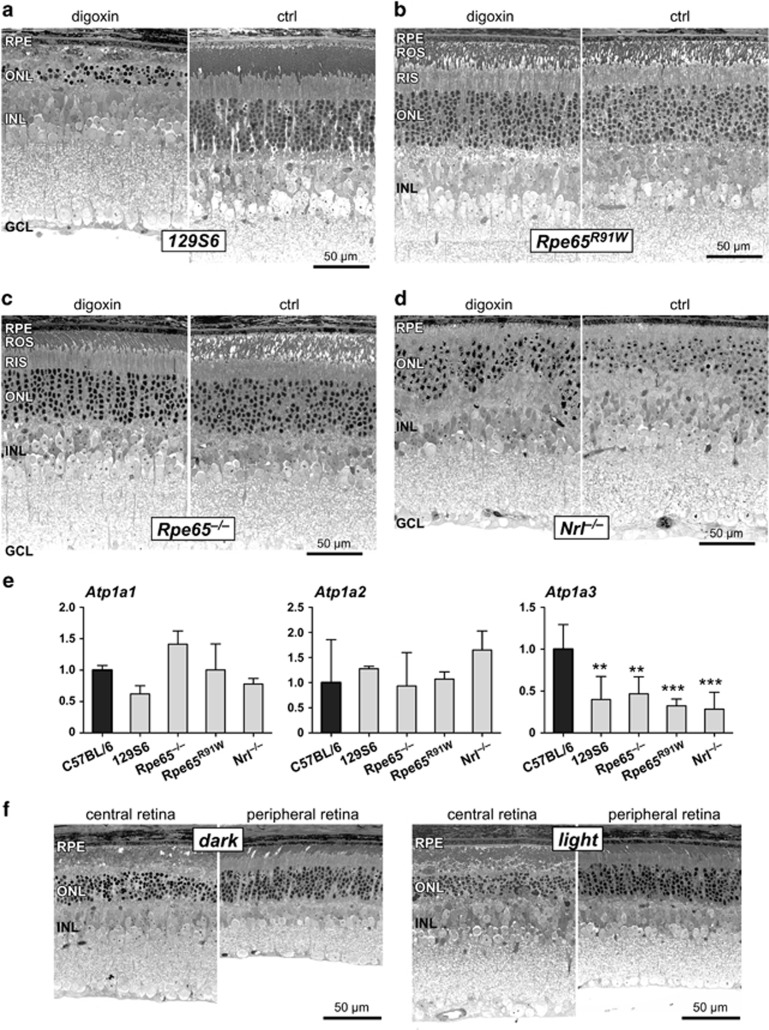
Dependency of digoxin-induced degeneration on rhodopsin. Retinal morphology of *129S6* wild type (**a**), *Rpe65*^*R91W*^ (**b**), *Rpe65*^*–/–*^ (**c**) and *Nrl*^*–/–*^ (**d**) mice at 2 days after the last of 3 injections of 2 mg/kg digoxin or of PBS (ctrl). *N*=3–4 (digoxin) and *N*=2–4 (ctrl). (**e**) Relative basal expression of *Atp1a1, Atp1a2* and *Atp1a3* in mice of the different strains as indicated. Expression levels were normalized to *Actb* and expressed relatively to levels in C57BL/6 mice (black bars, set to 1). Shown are means±S.D. of *N*=3. **: adjusted *P*-value<0.01. ***: adjusted *P*-value<0.001. *N*=3. One-way ANOVA with Dunnett's multiple comparison test (each column compared to C57BL/6). (**f**) Retinal morphology of *129S6* wild type at 2 days after the last of 3 injections of 2 mg/kg digoxin. Mice were either kept in darkness after the last injection according to the standard protocol (dark) or were exposed to a regular 12 h : 12 h light–dark cycle (light, 60 lux during the light period). *N*=3. Ctrl, PBS-injected control mice; GCL, ganglion cell layer; INL, inner nuclear layer; ONL, outer nuclear layer; RPE, retinal pigment epithelium; ROS, rod outer segments; RIS, rod inner segments; Scale bars, 50 *μ*m

As digoxin-induced retinal degeneration might be used as a model to study degenerative processes, we tested the susceptibility of different mouse strains to degeneration. Similar to C57BL/6 mice, photoreceptors of 129S6 wild-type mice were strongly damaged 2 days after the last injection of digoxin (3 × 2 mg/kg; Figure 6a). Surprisingly, however, photoreceptors of *Rpe65*^*R91W*^ mice on a 129S6 genetic background were completely insensitive to digoxin treatment (Figure 6b). Similarly, photoreceptors of *Rpe65*^*–/–*^ mice that are on a C57BL/6 background were also not damaged (Figure 6c). Common to these two mouse strains is the reduced amount (in *Rpe65*^*R91W*^) or lack (in *Rpe65*^*–/–*^) of bleachable rhodopsin caused by the low activity or complete absence, respectively, of RPE65 in the RPE.^33^ Furthermore, cone photoreceptors of *Nrl*^*–/–*^ mice did also not degenerate after digoxin application (Figure 6d). These data suggest that rhodopsin is required for digoxin-induced photoreceptor degeneration and that cones may be insensitive to digoxin cytotoxicity. Therefore, the influence on cone morphology (Figure 3) and function (Figures 4a and b) observed in C57BL/6 mice might have been indirectly caused by the degeneration of rods. Lack of digoxin sensitivity was not due to reduced expression of *Atp1a* genes in retinas of the respective strains since *Atp1a1* and *Atp1a2* were similarly expressed in all strains tested. Although the protected transgenic mice expressed *Atp1a3* at lower levels than C57BL/6 mice, expression was nevertheless similar to the susceptible mice of the 129S6 strain (Figure 6e).

As degeneration depended on rhodopsin, we tested whether increased photon absorption would accelerate the degenerative process in C57BL/6 mice. However, mice that were kept in cyclic light during treatment and during the 2-day post-treatment period (light) were damaged similarly to mice that were kept in constant darkness after the last digoxin injection (dark). The central retinal region showed similar levels of pyknotic photoreceptor nuclei and disrupted inner and outer segments whereas the peripheral retinal areas were spared independently of the light condition (Figure 6f).

## Discussion

We showed that daily intraperitoneal injections of 2 mg/kg digoxin on 3 consecutive days induced severe retinal degeneration in wild-type mice. Degeneration was restricted to photoreceptors of the central retina, depended on the presence of rhodopsin, activated stress signaling, and induced genes involved in degeneration, inflammation and oxidative stress. The dose needed to induce retinal degeneration was 100 to 1000-fold higher than doses used to treat heart conditions in human patients. Indeed, reports indicate that patients treated with a daily dose of as little as few *μ*g/kg digoxin may occasionally experience blurred vision, dyschromatopsia and other visual symptoms as adverse effects.^19^ Whether treatment also cause some photoreceptor toxicity in patients has not been investigated as most disturbances seem reversible at the dose used in clinics.^34^ In mice, however, significantly higher doses are required for an effect since polymorphisms in murine Na,K-ATPases make mice profoundly more resistant to digoxin.^20, 35, 36^

Digoxin was recently shown to inhibit HIF1 in the model of oxygen-induced retinopathy (OIR)^31, 32^ and suppressed choroidal neovascularization (CNV) in a model of laser-induced lesions.^32^ Although both studies used wild-type mice, a digoxin concentration of 2 mg/kg (as used here) and repetitive intraperitoneal injections, no cytotoxic effects on photoreceptors were reported. The reason for this is unclear but may be based on the age of the mice during treatment. Both OIR studies injected digoxin between PND12 and PND17, at a time when retinal development has not yet been completed. In fact, photoreceptor function is very low at least up to PND16^37^ suggesting that Na,K-ATPases may not yet be fully functional at this time. Although Yoshida *et al.*^32^ also used adult mice in their model of laser-induced CNV, they analyzed treatment outcome in choroidal flat mounts focusing on neovascularization. A specific effect on photoreceptors was not investigated in their experiments.

Digoxin inhibits primarily the catalytic alpha subunits of Na,K-ATPases^14^ and has a certain selectivity for α2 and α3 isoforms^7^ that are also expressed in retinal cells including photoreceptors.^17, 38^ As Na,K-ATPases are responsible for ion homeostasis and are critical for maintaining high extracellular levels of Na^+^ and high intracellular K^+^,^39^ inhibition of Na,K-ATPase activity by digoxin leads to an increase in Na^+^ and a depletion of K^+^ in the cells.^39, 40^ As a consequence of high intracellular Na^+^, the entry-mode of the sodium/calcium exchanger (NCX) may be activated^41^ potentially causing increased intracellular Ca^2+^ levels in addition.^42^ Whereas a causative role of high intracellular Na^+^ for the induction of cell death has not been established but may be connected to apoptosis through the regulation of cell volume,^43^ Ca^2+^ has long been recognized as an important factor for the regulation of a variety of cellular processes including apoptosis.^44^ Ca^2+^ may induce cell death, for example, through activation of calpains that leads to the proteolytic cleavage of intracellular substrates.^45^ Importantly, calpain-mediated cell death has also been reported in models of photoreceptor degeneration.^46, 47^ Similarly, strong evidence suggests that depletion of intracellular K^+^ is essential for the initiation of apoptosis in a variety of cells.^48^ The mechanisms that contribute to cell death by K^+^ depletion are not known in detail, but may include regulation of cell volume, caspase activation and changes of the membrane potential of mitochondria.^39^ Loss of intracellular K^+^ was directly implicated in photoreceptor degeneration in drosophila that lacked the alpha subunit of the Na,K-ATPase.^49^ Thus, inhibition of Na,K-ATPases may alter intracellular ion homeostasis, which may significantly contribute to the degeneration of photoreceptor cells after digoxin application.

Increasing evidence suggests that Na,K-ATPases not only control cellular ion homeostasis but have also important functions in signal transduction^50^ by interacting with endogenous cardenolides including digoxin-like substances that are found in the circulation.^51, 52^ Signaling may include activation of mitogen-activated protein kinases including ERK1/2,^53^ activation of PI3K and AKT^29^ and phosphorylation of SRC.^54^ Na,K-ATPases may thus constitute a class of cell surface receptors that can influence a number of physiological processes linked to cell survival in several cell types^50^ including neurons.^55^ We detected slight upregulation of pERK1/2 and pAKT at 1–2 days after digoxin injection. As digoxin has a half-life of ~40 h in patients,^56^ it is likely that digoxin can still signal at this time point in mice. However, as ERK1/2 and AKT are also activated by degenerative processes, we cannot discriminate between a direct digoxin-mediated effect and stress response signaling. It is interesting to note that both, pERK1/2 and pAMPK, levels drop below control levels late during the degenerative process. The reason for this is unclear but may signify the specific consequences of digoxin treatment.

Interestingly, photoreceptor degeneration caused by digoxin resembles light-induced retinal degeneration in several points. (i) degeneration is confined to photoreceptors^57^ (Figures 1 and 3); (ii) the central retina is more sensitive to damage^57^ (Figure 2); (iii) similar signaling pathways are activated^58, 59^ (Figure 5); (iv) cones are less sensitive^60^ (Figure 6); and (v) degeneration depends on rhodopsin^61, 62^ (Figure 6). The latter point is intriguing and not easily explained. Although hypotheses remain highly speculative, this result may indicate either an interaction of digoxin with rhodopsin inducing cytotoxicity by an unknown mechanism or an augmentation of digoxin-induced cell stress by light absorption, for example by generating all-trans-retinal as a reactive aldehyde^63^ or reactive oxygen species.^64^ However, digoxin-induced degeneration did not notably differ between mice that were kept in darkness or exposed to normal cycling light after digoxin treatment (Figure 6f), making the first or an alternative explanation more likely. In any case, both light exposure and digoxin application cause altered intracellular ratios of Na^+^/Ca^2+^/K^+^ through closure of the cGMP-gated channels or inhibition of Na,K-ATPases, respectively. This leads to degenerative processes with several common features suggesting that maintenance of ion homeostasis is a crucial determinant of photoreceptor survival. Studying this model in more detail may shed light onto the regulation of ion homeostasis in connection to physiologic (signaling) and pathologic (degeneration) processes. This model may also provide an alternative to the induction of photoreceptor apoptosis by the highly toxic MNU that was used frequently to investigate cellular and molecular aspects of photoreceptor degeneration.

## Material and methods

### Mice and digoxin injections

All animal experimentation was performed in accordance to the ARVO Statement for the use of animals in ophthalmic and vision research and the regulations of the veterinary authorities of Zürich. C57BL/6 (wild type; Jackson lab, Sulzfeld, Germany), 129S6 (wild type; Taconic, Ejby, Denmark), *Nrl*^*–/–*^,^65^ *Rpe65*^*–/–*^ (ref. 66) and *Rpe65*^*R91W*^ (ref. 33) mice were housed in the animal facility of the University of Zürich in a 12 h : 12 h light–dark cycle with access to food and water *ad libitum*. Three mice per strain and experimental condition were used at the age of 6–10 weeks, unless indicated otherwise.

Digoxin (Sigma, Buchs, Switzerland) was dissolved at a concentration of 40 mg/mL in DMSO, followed by dilution with PBS to 2 mg/mL and stored in aliquots at −20 °C. Immediately before use, digoxin was further diluted in PBS to 0.2 mg/ml and injected intraperitoneally at doses ranging from 0.1 mg/kg to 2 mg/kg. Injections were done once a day at 9–10 am for up to 3 consecutive days. Mice that were injected with PBS served as controls, except for Figure 2b where uninjected mice were used as controls. During digoxin treatment, mice were housed in normal cages in a 12 h : 12 h light–dark cycle (60 lux at cage level) with unrestricted access to food and water. After the last injection, mice were placed in darkness for 2 days before they were either killed or returned to the light–dark cycle until analysis. For the generation of data shown in Figure 6f (light), mice were kept in the normal light–dark cycle for the entire duration of the experiment.

### Morphology

Eyes were marked nasally, enucleated, fixed in glutaraldehyde (2.5% in cacodylate buffer) over night at 4 °C, trimmed, post-fixed in 1% osmium tetroxide and embedded in Epon 812 as described.^67^ Dorsoventral cross-sections of 0.5 *μ*m were cut through the optic nerve head, stained with toluidine blue and analyzed using light microscopy (Zeiss, Axioplan, Jena, Germany). Images of higher magnifications (Figures 1 and 6) were always acquired from the central region close to the optic nerve head, except where stated otherwise.

### Immunofluorescence and TUNEL staining

Eyes were marked nasally, enucleated and prepared for cryosectioning essentially as described^68^ but without prior perfusion of the mice. Cryosections (12 *μ*m) were cut in a dorsoventral orientation, blocked for 1 h with blocking solution containing 3% normal goat serum (Sigma) and 0.3% Triton X-100 (Sigma) in PBS. Sections were incubated over night at 4 °C with the following primary antibodies: PNA-FITC (1:250, L7381, Sigma), rabbit anti-GNAT1 (1:500, sc-389, Santa Cruz Biotechnology; Santa Cruz, CA, USA), mouse anti-GFAP (1:250, G3893-Clone G-A-5, Sigma), rabbit anti-IBA1 (1:500, 019-19741, Wako, Neuss, Germany), mouse anti-POU4F1 (alias BRN3A; 1:100, MAB1585, Chemicon, Temecula, CA, USA), rabbit anti-VSX2 (alias CHX10; 1:500, kindly provided by Connie Cepko, Harvard University, MA, USA), mouse anti-CR (1:1000, AB5054, Chemicon) and rabbit anti-CALB1 (1:500, AB1778, Chemicon). Sections were washed with PBS, incubated for 1 h at room temperature with appropriate secondary antibodies labeled with Cy2 or Cy3 (Jackson ImmunoResearch Laboratories, Soham, UK), counterstained with 4′,6′-diamidino-2-phenylindole (DAPI; Life Technologies, Zug, Switzerland) and analyzed using fluorescence microscopy (Zeiss).

For TUNEL staining, retinal cryosections were dried and fixed in 4% PFA solution for 10 min. After two washing steps with PBS (10 min each) a drop of 0.1% Triton X-100 in 0.1% sodium citrate was added and incubated for 5 min for tissue permeabilization. After two washing steps with PBS (5 min each) sections were tested for TUNEL-positive signals using the *in situ* cell death detection kit (Roche Diagnostics, Rotkreuz, Switzerland) according to the manufacturer's instructions. Slides were mounted with Mowiol (Sigma), sealed with nail polish solution and analyzed by fluorescence microscopy. Images were always acquired from the central region close to the optic nerve head.

### RNA isolation and semi-quantitative real-time PCR

Retinas were isolated through a slit in the cornea and snap frozen in liquid nitrogen. Retinal RNA was isolated using an RNA isolation kit according to the manufacturer's instructions (RNeasy, Qiagen, Hilden, Germany) with an additional on-column DNAse treatment to digest remaining genomic DNA. Concentration of eluted RNA was measured by Nanodrop spectrophotometer (Thermo Fisher Scientific, Waltham, MA, USA) and RNA diluted to a final concentration of 50 ng/*μ*l. cDNA was prepared from total RNA using oligo(dT) and M-MLV reverse transcriptase (Promega, Dübendorf, Switzerland). Semi-quantitative real-time PCR was performed in a LightCycler480 instrument (Roche Diagnostics) using the SYBR Green I master mix (Roche Diagnostics) and appropriate primer pairs (Table 1) designed to span large intronic regions and avoid known SNPs. Reactions were normalized to *Actb* and relative expression was calculated by the comparative threshold cycle method (ΔΔC_T_) using the LightCycler480 software.

### Protein isolation and Western blot

Retinas were isolated through a slit in the cornea and homogenized by sonication in 200 *μ*l of 100 mM Tris/HCl (pH 8,0). Homogenates were centrifuged (1000 × g; 3 min) and protein contents determined in the supernatants using Bradford reagent (Bio-Rad, Hercules, CA, USA). SDS–PAGE and Western blot analysis were performed as described^67^ using the following primary antibodies: rabbit anti-pERK (1 : 1000, #9101, New England Biolabs, Herts, UK), rabbit anti-ERK (1 : 1000, #9102, New England Biolabs), rabbit anti-pAKT_Ser473_ (1:1000, #9271, Cell Signaling Technology, Danvers, MA, USA); rabbit anti-pSTAT1_Tyr701_ (1:500, #9171, Cell Signaling Technology); rabbit anti-pSTAT3_Tyr705_ (1:500, #913 L, Cell Signaling Technology); rabbit anti-pAMPKa1/2_Thr172_ (1:1000, sc-33524, Santa Cruz Biotechnology); rabbit anti-CASP1 (1:10 000, kindly provided by P. Vandenabeele, University of Gent, Gent, Belgium); mouse anti-GFAP (1:1000, G3893-Clone G-A-5, Sigma); rabbit anti-HIF-1 A (1:4000, NB100-479, Novus Biologicals, Cambridge, UK); rabbit anti-PI3K (1:4000, D0669, Upstate, Millipore, Darmstadt, Germany); mouse anti-ACTB (1:10,000, A5441, Sigma). Membranes were incubated with primary antibodies diluted (as indicated) in 5% non-fat blocking milk (Bio-Rad) in TBST over night at 4 °C with gentle agitation. After a 1-h incubation step with appropriate HRP-conjugated secondary antibodies, immunoreactive signals were detected using the Western lightning chemiluminescence reagent (PerkinElmer, Waltham, MA, USA). Signals were visualized using X-ray films.

### Electroretinography, fundus imaging and OCT

Mice were dark adaptated over night and pupils dilated using Cyclogyl 1% (Alcon Pharmaceuticals, Fribourg, Switzerland) and Neosynephrine 5% (Ursapharm Schweiz GmbH, Roggwil, Switzerland) 20 min before recording. Mice were anesthetized by a subcutaneous injection of ketamine (85 mg/kg, Parke-Davis, Berlin, Germany) and Xylazine (4 mg/kg, Bayer AG, Leverkusen, Germany). A drop of mydriacticum dispersa (OmniVision AG, Neuhausen, Switzerland) was applied to each cornea to induce mydriasis and to keep the tissue moist. Recordings were done using flashes of 13 different light intensities ranging from −50 db (0.000025 cd*s/m^2^) to 15 db (79 cd*s/m^2^) for scotopic and flashes of 8 different light intensities ranging from −10 db (25 cd*s/m^2^) to 25 db (790 cd*s/m^2^) for photopic ERG as described.^69^ Ten recordings were averaged per light intensity.

Fundus imaging and OCT were done essentially as described.^70^ In brief, pupils of mice were dilated and mice anesthetized as describe as above. Eyes were kept moist with 2% methocel (OmniVision AG) and fundus images and OCT scans recorded using the Micron IV system (Phoenix Research Labs, Pleasanton, CA, USA).

### Statistical analysis

Differences in gene expression levels were evaluated by one-way ANOVA followed by Dunnett's multiple comparison tests (GraphPad, San Diego, CA, USA) comparing all time points (Figures 5a and b) or strains (Figure 6e) to the control or C57BL/6, respectively.

## Figures and Tables

**Table 1:** Primers used for real-time PCR

**Gene**	**Forward 5′–3′**	**Reverse 5′–3′**	**Product (bp)**
*Actb*	CAACGGCTCCGGCATGTGC	CTCTTGCTCTGGGCCTCG	153
*Atp1a1*	GGTGGTGCTCTCTGCTGTAG	GACGACATCCTCCGCATTGA	161
*Atp1a2*	CTGTCCTTGGATGAGCTGGG	CCTGAGCTCGCTGATTGGTG	72
*Atp1a3*	GAACTTCACCACAGACAACC	GACCATGATGACCTTGATGC	121
*Edn2*	AGACCTCCTCCGAAAGCTG	CTGGCTGTAGCTGGCAAAG	64
*Fgf2*	TGTGTCTATCAAGGGAGTGTGTGC	ACCAACTGGAGTATTTCCGTGACCG	158
*Casp1*	GGCAGGAATTCTGGAGCTTCAA	GTCAGTCCTGGAAATGTGCC	138
*Tnf*	CCACGCTCTTCTGTCTACTGA	GGCCATAGAACTGATGAGAGG	95
*Hmox1*	CCGCCTTCCTGCTCAACATT	GACGAAGTGACGCCATCTGTG	99
*Gfap*	CCACCAAACTGGCTGATGTCTAC	TTCTCTCCAAATCCACACGAGC	240
